# Supplementary material for: A temporal sequence of heterochronic gene activities promotes stage-specific developmental events in Caenorhabditis elegans
Source: G3 (Bethesda). 2024 Jun 12;14(8):jkae130. doi: 10.1093/g3journal/jkae130 (PMC11304605; doi:10.1093/g3journal/jkae130)
Supplement: jkae130_Supplementary_Data [file jkae130_supplementary_data.docx]

## Supplementary information

### Table S1 List of experiments. Strains in each experiment were synchronized at the same time and transfers were carried out at similar time points.

| Expt. number | Strains synchronized | Direction of transfers | Hours after synchronization |
| --- | --- | --- | --- |
| 1 | *lin-14::AID, lin-28::AID* | To 5-Ph-IAA | 2, 4, 6, 8, 10 |
| 2 | *lin-14::AID, lin-28::AID* | To 5-Ph-IAA | 12, 14, 16, 18, 20 |
| 3 | *lin-14::AID* | To 5-Ph-IAA | 8, 10 |
| 4 | *lin-28::AID, hbl-1::AID* | To 5-Ph-IAA | 14, 16, 18, 20, 22, 24 |
| 5 | *lin-28::AID, hbl-1::AID* | To 5-Ph-IAA | 24, 26, 28, 30, 32 |
| 6 | *lin-14::AID* | To 5-Ph-IAA | 13, 14, 16, 18, 20, 22 |
| 7 | *hbl-1::AID, lin-41::AID* | To 5-Ph-IAA | 24, 26, 28, 30, 32 |
| 8 | *hbl-1::AID, lin-41::AID* | To 5-Ph-IAA, Off 5-Ph-IAA | 12, 16, 18, 20, 22, 36, 38, 40 |
| 9 | *hbl-1::AID, lin-41::AID* | To 5-Ph-IAA, Off 5-Ph-IAA | 8, 24, 28, 32, 34 |
| 10 | *hbl-1::AID* | To 5-Ph-IAA, Off 5-Ph-IAA | 14, 16, 18, 20, 22, 24 |
| 11 | *hbl-1::AID* | To 5-Ph-IAA, Off 5-Ph-IAA | 24, 26, 28, 30 |
| 12 | *lin-14::AID* | To 5-Ph-IAA, Off 5-Ph-IAA | 1, 2, 4, 6, 8 |
| 13 | *lin-41::AID* | To 5-Ph-IAA | 36, 38, 40, 42, 44 |
| 14 | *lin-41::AID* | Off 5-Ph-IAA | 40, 42 |

##

## Table S2. Degron insertion sequences. Auxin-inducible degron sequence is in bold.

| *lin-14::AID* — degron at 3’ end of ORF; altered *lin-14* sequence in lowercase; stop is underlinedATCACAATCTCCcCCgttaCAgGGaCCcCAgA**TGCCTAAAGATCCAGCCAAACCTCCGGCCAAGGCACAAGTTGTGGGATGGCCACCGGTGAGATCATACCGGAAGAACGTGATGGTTTCCTGCCAAAAATCAAGCGGTGGCCCGGAGGCGGCGGCGTTCGTGAAG**TAGCAAGGTCCACAATAG |
| --- |
| *lin-28::AID* — degron at 3’ end of ORF; altered *lin-28* sequence in lowercase; stop is underlinedATCATCACCGACgACgTCggatgacGAcATccGAGAgAAaAATAGcAATTCaTCcGAcGAg**ATGCCTAAAGATCCAGCCAAACCTCCGGCCAAGGCACAAGTTGTGGGATGGCCACCGGTGAGATCATACCGGAAGAACGTGATGGTTTCCTGCCAAAAATCAAGCGGTGGCCCGGAGGCGGCGGCGTTCGTGAAG**TAGACATCTGACGATGAT |
| *hbl-1:AID* — degron at 3’ end of ORF; altered *hbl-1* sequence in lowercase; stop is underlinedTGCTCTCCACATGTAtCAgGCgcGtCAtCAg**ATGCCTAAAGATCCAGCCAAACCTCCGGCCAAGGCACAAGTTGTGGGATGGCCACCGGTGAGATCATACCGGAAGAACGTGATGGTTTCCTGCCAAAAATCAAGCGGTGGCCCGGAGGCGGCGGCGTTCGTGAAG**TAAGTACCAAGCCAGACACCAATAATGAGGACGTCCTCGTTAA |
| *lin-41::AID* — degron at 5’ end of ORF; start is underlined ATGGCGACCATCGTG**ATGCGTAAAGATCCAGCCAAACCTCCGGCCAAGGCACAAGTTGTGGGATGGCCACCGGTGAGATCATACCGGAAGAACGTGATGGTTTCCTGCCAAAAATCAAGCGGTGGCCCGGAGGCGGCGGCGTTCGTGAAG**ATGGCAACGATTGTC |

##


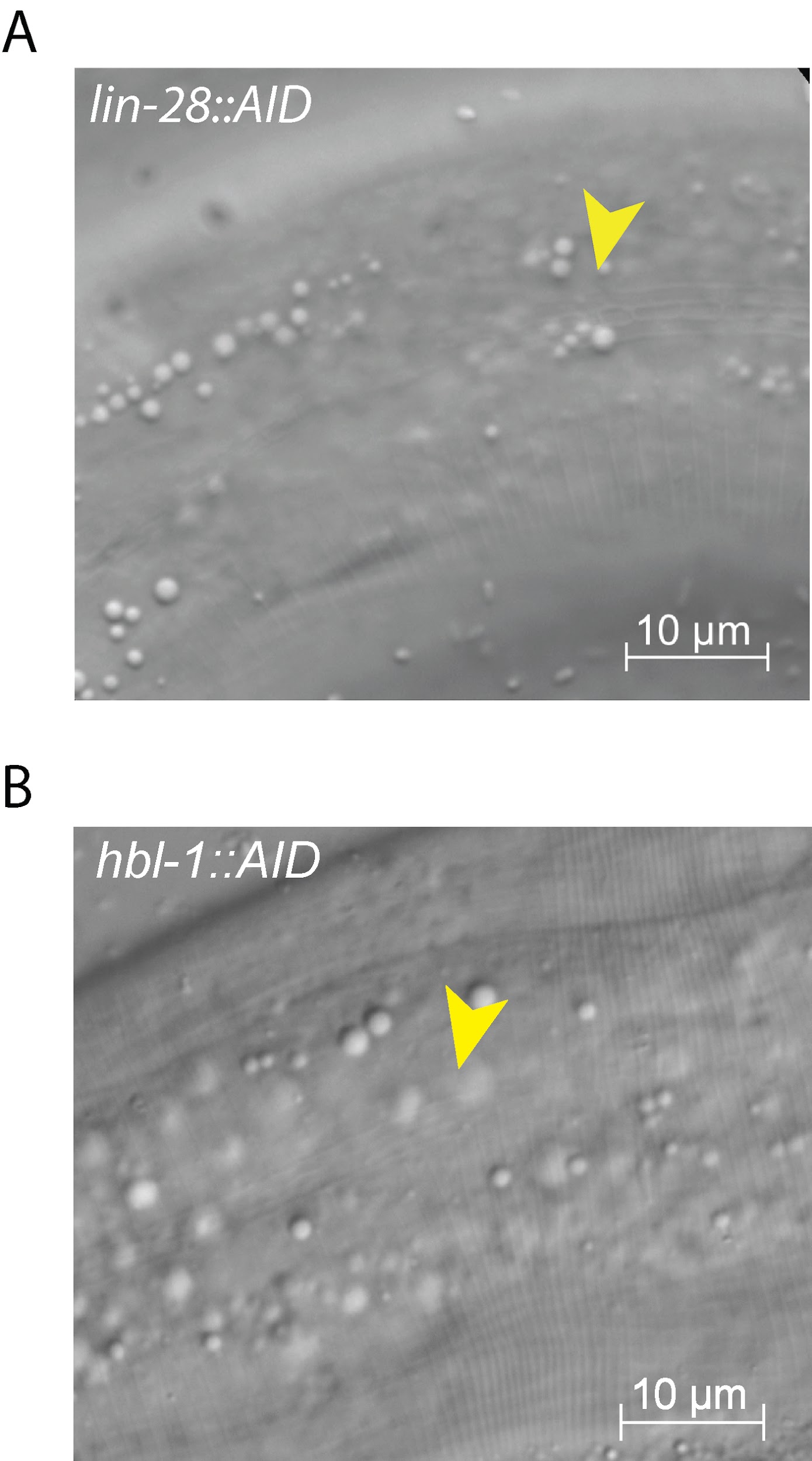


### Fig. S1. Precocious alae appearances differ in *lin-28::AID* and *hbl-1::AID* animals on 5-Ph-IAA. DIC microphotographs of L4 animals grown at 20°C. Animals are oriented anterior to the left, dorsal side up. (A) *lin-28::AID* animals develop clear alae patches when moved to 5-Ph-IAA at times close to the end of the second activity. The yellow arrowhead indicates the edge of an alae patch. (B) *hbl-1::AID* animals might develop thin and transparent alae when moved to or away from 5-Ph-IAA at times close to the end of the second activity, the edges of alae patches are hard to discern. The animal on the image was moved away from 5-Ph-IAA at 21 hours in development. The yellow arrowhead indicates alae.
